# Supplementary material for: Cavity-enhanced single-shot readout of a quantum dot spin within 3 nanoseconds
Source: Nat Commun. 2023 Jul 5;14:3977. doi: 10.1038/s41467-023-39568-1 (PMC10322905; doi:10.1038/s41467-023-39568-1)
Supplement: Supplementary file 1 — Supplementary Information [file 41467_2023_39568_MOESM1_ESM.pdf]

Supplementary Information: Cavity-enhanced single-shot readout of a quantum dot spin within 3 nanoseconds

Supplementary Note 1. EXTRACTION OF SPIN LIFETIME FROM $g^{(2)}(\tau)$

We can characterise the spin-flip rate in our experiment by measuring the second-order correlation function of the resonance fluorescence $g^{(2)}(\tau)$. By driving only one of the Zeeman-split trion states, spin-flips are observed as blinking in the quantum dot (QD) fluorescence as the spin state switches between the on-resonance (bright) and off-resonance (dark) states. This blinking results in bunching of the $g^{(2)}(\tau)$-function [1]. This blinking is not present at zero magnetic field, where the trion states are degenerate such that both spin states are driven with a resonant linearly-polarised laser. Supplementary Fig. 1(a) shows $g^{(2)}(\tau)$ measured at zero magnetic field for the X^- transition of the QD used in our experiments. As expected for a single emitter, anti-bunching is observed at $\tau = 0$. Away from $\tau = 0$, the $g^{(2)}(\tau)$ is flat with no significant bunching observed, indicating that the QD emission is stable.

Supplementary Fig. 1 (b) shows a similar $g^{(2)}(\tau)$-measurement, now for an out-of-plane magnetic field $B = 2.0\,$T. Here, the laser drives the higher-frequency trion state, and clear bunching is observed. By fitting the measured $g^{(2)}(\tau)$ to an exponential decay, we can extract the characteristic timescale on which the QD emission switches on and off. Because the switching between bright and dark states occurs purely due to spin flips, the timescale of the bunching decay is a direct measurement of the spin lifetime, τ_{SF} [2].

We measured the $g^{(2)}(\tau)$ and extracted τ_{SF} for several different positions on the X^- charge plateau. The results are summarised in Supplementary Fig. 2, where the red stars indicate the charge plateau position at which each experiment was performed. The data in Supplementary Fig. 2 were acquired using low laser powers, significantly below the saturation power. The spin lifetime is very short, a few nanoseconds, at the edges of the charging plateau, and reaches a modest value, $\sim 140\,$ns, at the centre of the plateau. These are the hallmarks of co-tunneling [3, 4], a process in which a combined tunnelling process swaps an electron confined to the QD with an electron close to the Fermi energy in the Fermi sea. From the measured spin lifetimes, it is clear that co-tunneling determines the spin lifetime even at the centre of the plateau. The observed maximum spin lifetime of 144 ns is orders-of-magnitude less than the expected intrinsic spin lifetime via a phonon-mediated process at this magnetic field: previous experiments using InGaAs QDs have demonstrated $\sim$ 20 ms at similar magnetic field strengths [4, 5]. The relatively fast co-tunneling is a consequence of the 25 nm-thick tunnel barrier, the distance separating the back contact and QD-layer in the heterostructure.

We note that due to the high speed at which we can perform single-shot spin readout, the relatively short co-tunneling induced spin-flip time that we observe is not the limiting factor for the readout fidelity. For a readout time of 3 ns, we would expect a spin flip during the readout pulse in only $1 - \exp(-3/144) \sim 2\%$ of readout attempts.

For the low powers used in Supplementary Fig. 2, we do not observe spin pumping, for which the typical signature is

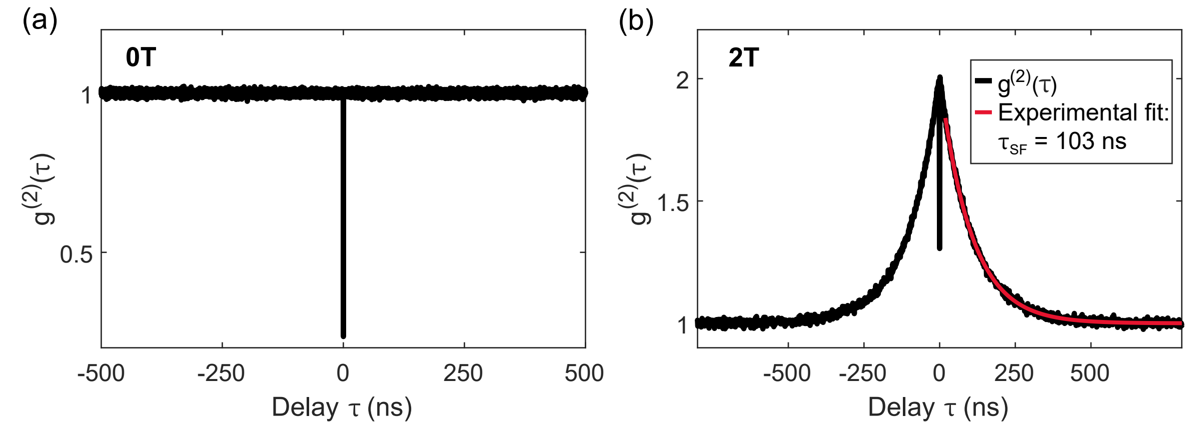

SUPPLEMENTARY FIG. 1: **Second-order correlation function at 0 T and 2 T. (a)** At $B = 0\,$T the $g^{(2)}(\tau)$ features anti-bunching at $\tau = 0$ but no bunching out to $\tau = 500\,$ns, demonstrating that the QD emission is stable on this timescale. **(b)** $g^{(2)}(\tau)$ at $B = 2\,$T while resonantly driving the higher-frequency trion state. The QD emission shows clear bunching. We fit the $g^{(2)}(\tau)$ with an exponential decay to determine the spin-flip rate. The data shown here is acquired with a laser power equal to the QD saturation power; from the fit we extract $\tau_{\mathrm{SF}} = 103\,$ns.

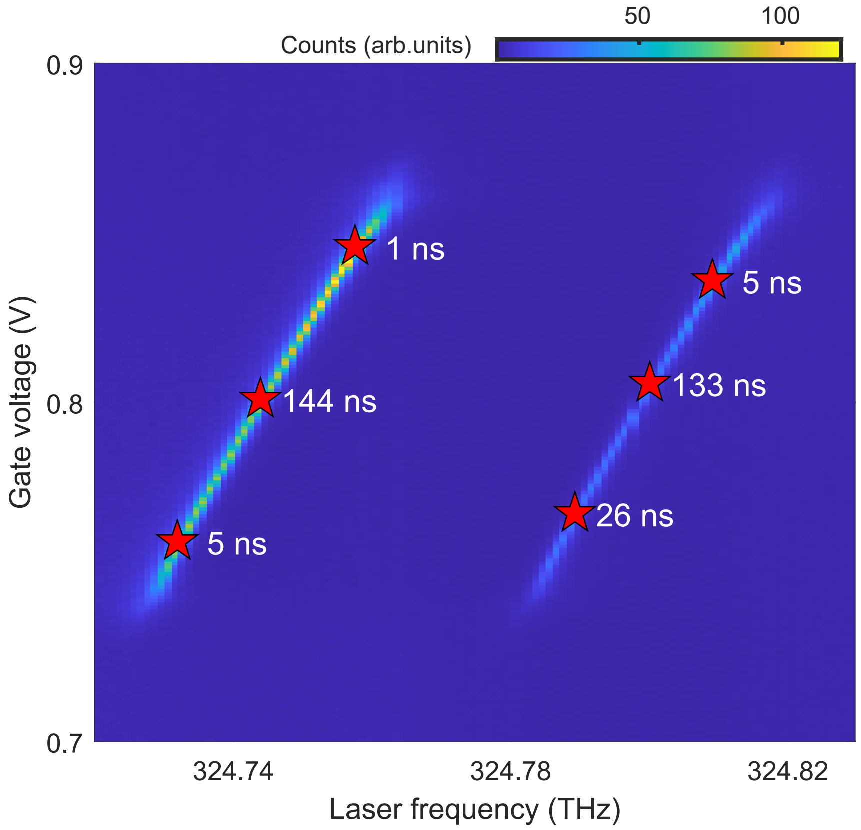

SUPPLEMENTARY FIG. 2: **Spin lifetime as a function of position within the charge plateau.** To map out the charge plateaus, the QD fluorescence was collected as a function of excitation laser frequency and gate voltage applied across the diode structure. The applied magnetic field is 2.0 T, resulting in a splitting of $\sim$ 55 GHz between the two vertical optical transitions. The red stars indicate positions on the Zeeman-split plateaus for which the spin lifetime was measured (as described in Supplementary Fig. 1(b)). The spin lifetime decreases at the plateau edges to very small values, a clear sign of co-tunnelling. However, even in the plateau centre the longest spin lifetime time we observe is 144 ns, also determined by co-tunnelling. We note that to acquire this data we adjust the cavity length when the laser frequency is stepped such that the laser remains on resonance with the cavity.

a region of decreased signal at the centre of the charge plateau: in spin pumping, the excitation results in occupation of the dark spin state [6, 7]. The absence of a spin pumping signature in Supplementary Fig. 2 indicates that the spin pumping rate is significantly smaller than the spin flip rate. Spin pumping arises via spin-nonconserving spontaneous emission, a diagonal transition, main text Fig. 2(b). (The spin-conserving recombination is the vertical transition, main text Fig. 2(b).) The branching ratio is the ratio of the diagonal to vertical recombination times. It can be inferred from the $g^{(2)}(\tau)$ recorded with optical driving powers above saturation power. Specifically, the branching ratio can be extracted from $g^{(2)}(\tau)$ by solving the incoherent part of the optical Bloch equations, i.e. the rate equations describing the populations of the three relevant QD levels [8]. Following this process, we extract a branching ratio of $\Gamma_s/\gamma_d = 600 \pm 200$ (where Γ_s is the vertical spin-conserving decay rate, and γ_d the diagonal spin-nonconserving rate). This branching ratio applies to the experimental conditions for the readout process in which one of the vertical transitions is in resonance with the cavity.

Spin-nonconserving spontaneous emission is the origin of back-action in the spin readout process. For single-shot readout, the branching ratio must be high enough for the spin state to be assigned with high fidelity before a laser-induced spin-flip transition occurs. This is the case here. In fact, the branching ratio is sufficiently high that back-action is a negligible source of readout error in these experiments.

Supplementary Note 2. SPIN INITIALISATION BY OPTICAL PUMPING

One strategy for demonstrating single-shot spin readout is to first initialise the spin in a known state, then perform the readout sequence. By comparing the spin state attributed during readout with the initially prepared state, the readout fidelity can be quantified. This method relies on the ability to initialise the spin state with high fidelity. In our experiments, the combination of a modest spin lifetime together with a large branching ratio make the initialisation of a known spin state via optical pumping challenging. The maximum spin pumping rate that we were able to achieve was comparable to the co-tunnelling rate in the centre of the plateau. Supplementary Fig. 3(a) shows a plateau map similar to Supplementary Fig. 2 using a significantly higher excitation laser power (approximately twice the saturation power). We now observe decreased fluorescence intensity in the plateau centre, consistent with spin pumping. However, rather than near-complete extinction of the fluorescence, the signal at the plateau centre is reduced by only a factor of about two (Figs. 3(b),(c)) compared to at the edges where rapid co-tunnelling prohibits spin pumping. The incomplete suppression of fluorescence in the centre of the charge plateau indicates that our spin initialisation fidelity using optical spin pumping is likely to be modest.

To quantify the achievable spin initialisation fidelity, we measured the spin-flip rate near the centre of the charge plateau as a function of laser power. The result is shown in Supplementary Fig. 3 (d); the two curves show τ_{SF} for the laser resonant with the high-frequency trion transition or the lower-frequency trion transition.

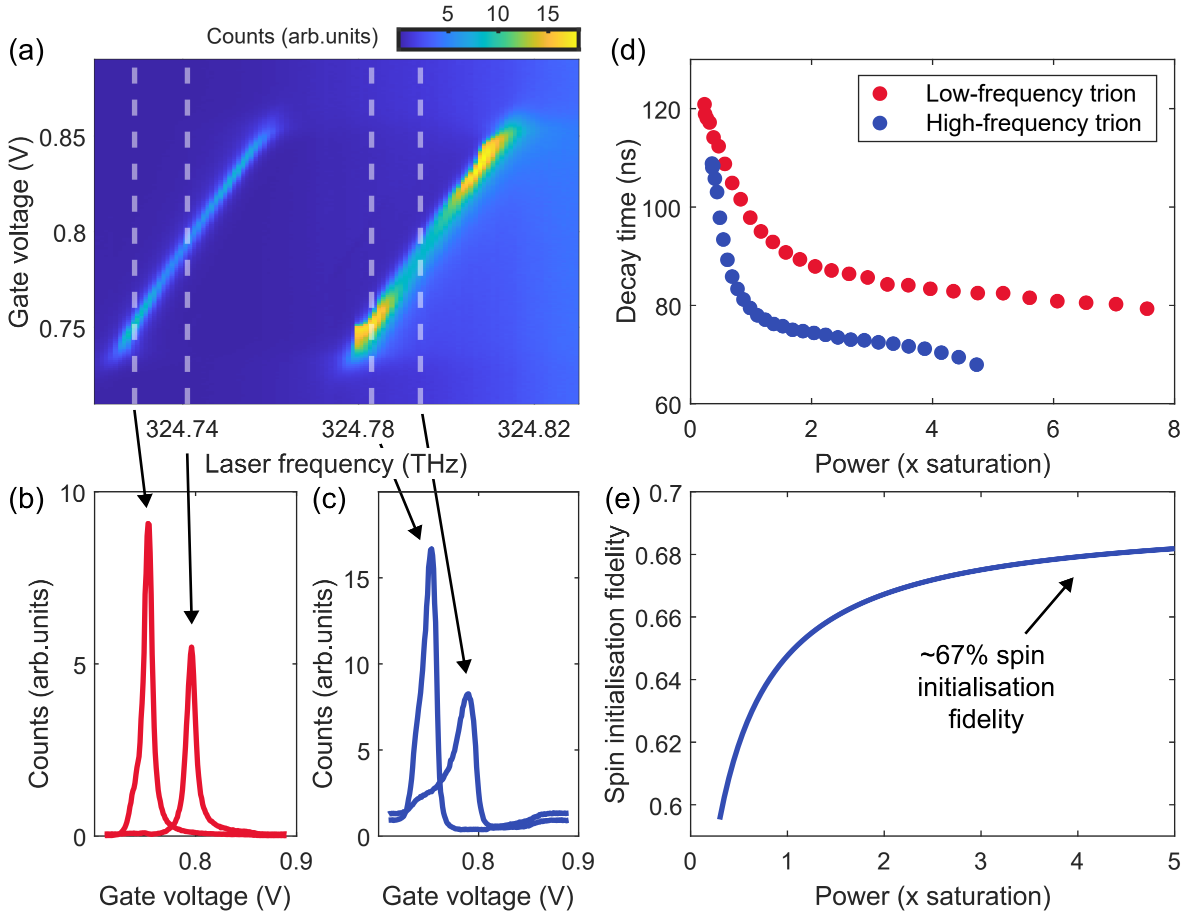

SUPPLEMENTARY FIG. 3: **Partial optical spin initialisation.** **(a)** Plateau map acquired with a laser power twice the saturation power. We observe a reduction in signal at the plateau centre, consistent with optical spin pumping. However, comparing the signal in the centre of plateau with that at the edges in the fast co-tunnelling regime suggests a poor spin initialisation fidelity. **(b)** Comparison of fluorescence signal for the low-frequency trion in the fast co-tunnelling regime (left, lower gate voltage) and in the plateau centre (right, higher gate voltage). **(c)** Similar to (b) but for the high-frequency trion. **(d)** Spin-flip times as a function of excitation laser power measured at the centre of the charge plateau, resonantly driving the low-frequency trion (blue data points) or the high-frequency trion (red data points). **(e)** Estimated spin initialisation fidelity from solving the incoherent part of three-level optical Bloch equations, using spin-flip rates extracted from (d). Based on this analysis, a maximum spin initialisation fidelity of $\sim 67\%$ is expected.

We solve the incoherent part of the optical Bloch equations to estimate the population of the spin initialisation target state (equivalent to the initialisation fidelity) as a function of the initialisation laser power, shown in Supplementary Fig. 3 (e). We find that the achievable initialisation fidelity saturates to a rather low value; for a laser power of 4x the QD saturation power, the initialisation fidelity (defined as $|\langle\psi_{\mathrm{actual}}|\psi_{\mathrm{target}}\rangle|^2$) is approximately 67%.

With such a low initialisation fidelity, a measurement sequence of first initialising the spin before readout is impractical, as the initialisation fidelity would dominate the total sequence fidelity and obscure the actual readout error. Instead, to characterise our single-shot readout fidelity we repeat our readout sequence with a delay comparable to the plateau-centre spin lifetime, which results in an approximately 50:50 spin state occupation probability over the course of a large number of sequential readout sequences. As discussed in Supplementary Note 5, by characterising each readout error process individually we can determine the overall readout fidelity.

We stress that the inability to initialise the spin with high fidelity in these experiments is a consequence of the tunnel barrier thickness and does not represent a limitation of the scheme itself. High initialisation fidelities can be achieved by suppressing the co-tunneling at the plateau centre using a larger tunnel barrier [6].

Supplementary Note 3. CONTINUOUS WAVE QUANTUM JUMPS MEASUREMENTS

Complementary to the experiments demonstrating quantum jumps using rapidly repeated readout pulses shown in Fig. 3(b) of the main text, we also observed quantum jumps using continuous wave (CW) excitation. A CW laser set to four times the saturation power of the bright state transition was used, and the emitted photons were routed (via cascaded 50:50 beam splitters) to four SNSPD detectors. In contrast to the pulsed single-shot readout experiments in the main text (where only one SNSPD detector was used), we used four detectors to mitigate partially the impact of the detectors' dead time. We note that the addition of the cascaded beam splitters reduces the overall system efficiency, hence why we focused on the pulsed quantum jumps experiments in the main text. We measured the signal on all four detectors simultaneously, and the resulting counts registered by the four detectors were then added together. If at least one photon is measured in a time-bin, the state is assigned spin up ($|\Uparrow\rangle$). If no photon is detected, the state is assigned spin down ($|\Downarrow\rangle$). A fraction of these quantum jumps is shown in Supplementary Fig. 4(a).

The CW quantum jumps we observe provide an additional method to characterise the spin-flip rates in our system, as $\tau_{\rm SF}$ can be directly extracted from the waiting-time distributions for $|\Uparrow\rangle$ and $|\Downarrow\rangle$. The time over which the spin state remains the same is extracted over an experiment of 50 ms duration, and its distribution is shown as a histogram in Supplementary Fig. 4(b). By fitting the decay in the histogram we determine a spin-flip time of $\tau_{\rm SF}$ = 109.9.9±4.1 ns. The result is slightly lower (although broadly consistent) with the spin-flip time extracted from the $g^{(2)}(\tau)$ recorded using the same laser power (Supplementary Note 1); the present experiment was performed slightly offset from the exact charge plateau centre, which may explain the difference. Due to partial spin-pumping with CW excitation (see Supplementary Note 2), the observed spin-flip time is slightly smaller than that measured with pulsed excitation (165 ns, main text).

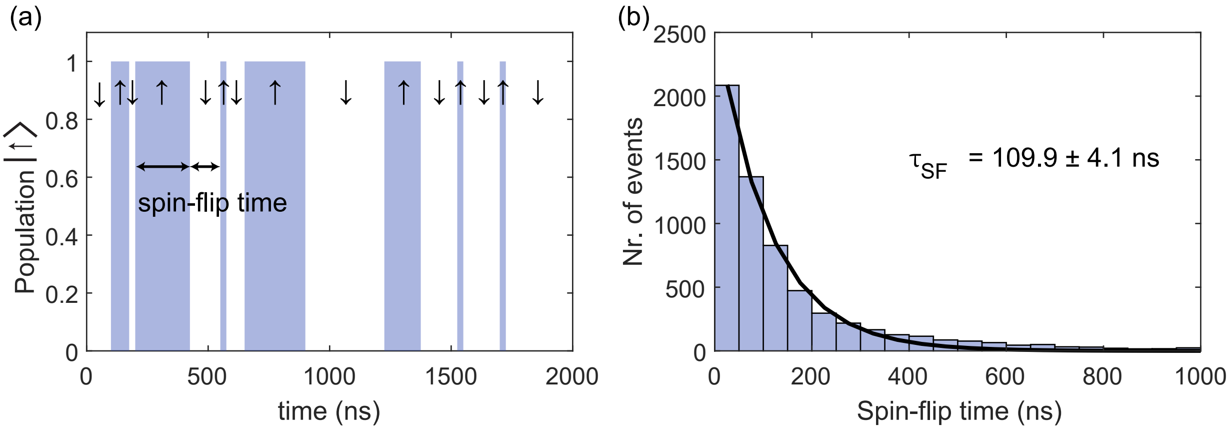

SUPPLEMENTARY FIG. 4: **CW Quantum Jumps (a)** Normalised photon counts as a function of detection time. The quantum jumps between the two spin states can be observed and the time over which the spin remains the same can be extracted. **(b)** Histogram of the extracted times between spin-flip events reveals an exponential decay. The spin-flip time is extracted from an exponential fit to be (109.9±4.1) ns.

Supplementary Note 4. CAVITY SUPPRESSION AT 2.0 T

In order to readout the spin-state correctly, the excitation laser has to be suppressed well enough to minimise spurious counts on the detector due to laser leakage. Otherwise, there is a significant probability that the readout pulse projects the spin into the dark spin-state yet the outcome is recorded falsely as the bright spin-state. A cross-polarisation setup is used to prevent laser light from entering the detection fibre [9]. This works extremely well at zero magnetic field. However, in an applied magnetic field, the background suppression works slightly less well. This effect likely arises from a Faraday effect in the top mirror of the cavity and/or objective lens. A normalised background signal showing the counts due to laser leakage as a function of cavity detuning is shown in Supplementary Fig. 5(a). Unfortunately, the point of maximum laser suppression is not aligned with the cavity resonance, but detuned by 7.5 GHz. At the cavity resonance, the background is high enough to give a spurious count on the detector in 80 % of the readout pulse repetitions, making this regime impractical for the readout. The 2 T measurements are therefore performed at the cavity detuning where the background is a minimum. At this cavity detuning, the probability of detecting a photon via laser leakage reduces to 1.4% for a 3 ns readout pulse.

An important parameter for the spin-readout is the β-factor which itself depends on the Purcell factor, F_P: $\beta = F_P/(F_P+1)$. Cavity-enhanced spin readout depends on achieving β-factors as close as possible to one, equivalently large Purcell factors. We extract the Purcell-factor as a function of cavity detuning by measuring the lifetime of the QD at each cavity detuning and deriving it via $\Gamma = F_P \cdot \gamma$, where Γ is the Purcell-enhanced decay rate and γ is the bare decay rate ($\gamma \approx 0.3$ GHz). (The decay rate is the inverse of the lifetime, $\gamma = 1/\tau$). On resonance with the cavity, $F_P = 8.5$. At the detuning for which the laser suppression works best, the Purcell factor is slightly lower, $F_P = 6.1$. The corresponding decay curves following excitation with a few-ps laser pulse are shown in Supplementary Fig. 5(b). The spin read-out experiments were carried out at $F_P = 6.1$.

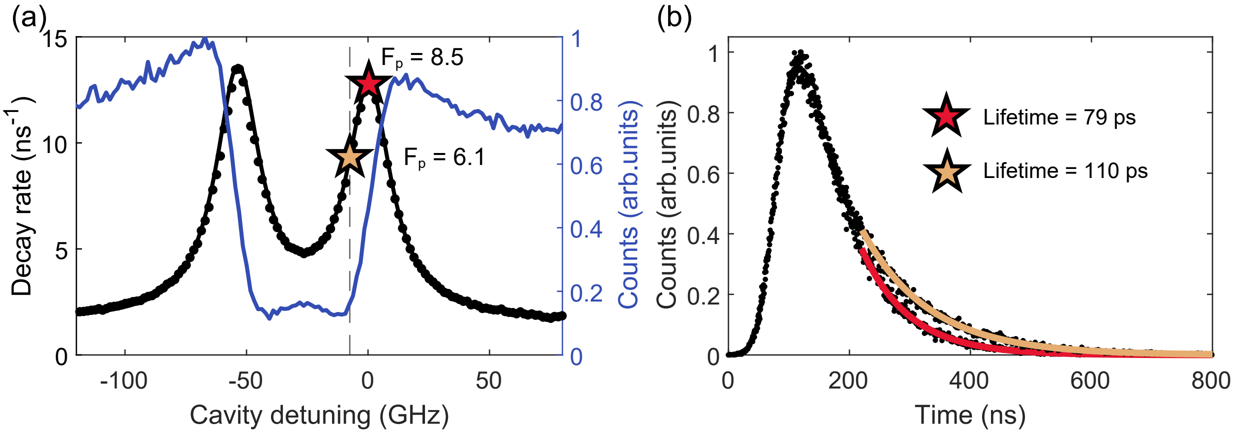

SUPPLEMENTARY FIG. 5: **(a)** Laser leakage into the collection channel (blue) and decay rate of the QD (black) as a function of cavity detuning. The laser leakage shows two minima and neither aligns perfectly with the cavity resonance. At the cavity resonance, the Purcell factor is $F_P = 8.5$; at the lowest laser leakage, $F_P = 6.1$. **(b)** Time-resolved lifetime measurement on resonance with the cavity (red) and at the detuning for minimum laser leakage (yellow). The response of the QD to a short excitation pulse (few ps) is measured and reveals an exponential decay (black). The lifetime is extracted from an exponential fit and is 79 ps at resonance and 110 ps at minimum laser leakage.

Supplementary Note 5. MONTE-CARLO SIMULATION: COUNT-TRACES AND FIDELITY

Our single-shot readout results are modelled using a Monte-Carlo approach in order to determine the readout fidelity.

Supplementary Note 5.1. Simulation of Readout Count fractions

The simulations of the count fractions are based on a Monte-Carlo method in which the (simulated) readout outcome is recorded many times (100,000 repetitions) in order to mimic the experiment. The readout pulse is considerably longer than the Purcell-enhanced radiative lifetime. The power is also well above the saturation power. These two factors mean that should the spin be projected into the bright state, the exciton population is close to 0.5. The photon emission rate is the occupation of the bright state divided by the lifetime. Each photon is detected with a certain probability, the overall system efficiency. In other words, the detection rate is the emission rate multiplied by the overall system efficiency, η. A readout cycle is repeated until a photon is detected and the detection time is recorded. Summing up over all repetitions leads to count fractions as in the measurements in Fig. 2(a) of the main text. The model has four input parameters: the overall system efficiency η (the probability that an exciton in the QD results in a click on the detector), the Purcell factor F_P, the spin-flip time τ_{SF}, and the probability of detecting a laser photon (to simulate the laser background, see analysis in Supplementary Note 4).

The dependence of the readout on the overall system efficiency is shown in Supplementary Fig. 6(a). The higher the efficiency, the sooner the spin-state can be read out, and the lower the probability of incorrectly assigning the spin state. In practice, the overall system efficiency η is known based on the analysis in Ref. [10], the properties of the fibre couplers, and the quantum efficiency of the detector:

$$\eta = \beta \cdot \frac{\kappa_{\mathrm{top}}}{\kappa + \gamma} \cdot \eta_{\mathrm{optics}} \cdot \eta_{\mathrm{coupler}} \cdot \eta_{\mathrm{detector}} \tag{1}$$

where β is the probability that an exciton creates a photon in the H-polarised cavity mode; $\kappa_{\mathrm{top}}/(\kappa + \gamma) = 96\%$ is the probability that a photon in the cavity exits the top mirror; and $\eta_{\mathrm{optics}} = 69\%$ represents the throughput of the optical system from microcavity to the output of the final output fibre (as defined and measured in Ref. [10]). The output of this fibre is coupled to the detector with an optical coupler (in practice, two fibre-couplers) with efficiency $\eta_{\mathrm{coupler}} = 80\%$. Finally, the detector has a quantum efficiency of $\eta_{\mathrm{detector}} = 82\%$.

At $B = 0$, $\beta = 86\%$ such that $\eta = 37\%$. We stress that this is the predicted overall system efficiency based on the analysis of all the individual contributions, including the detector efficiency. In practice, this predicted value of η describes the experimental results extremely well.

At $B = 2\,\mathrm{T}$, $\beta = 80\%$, resulting in a predicted overall efficiency of $\eta = 35\%$. In practice, a slightly lower η is required to describe quantitatively the experimental results, $\eta = 25\%$. The origin of this slight reduction in η with respect to $B = 0$ is unknown. For the cavity alignment with $\mathrm{F_p} = 6.1$ used in our experiments (shown in Supplementary Fig. 5 (a)) the Purcell factor is very sensitive to the exact cavity detuning; a small shift could result in a slightly lower Purcell factor. The effect of a lower Purcell factor in our simulations is similar to that of a lower efficiency. Other explanations could be a deterioration in either the in-coupling efficiency (such that the power exceeds the saturation power by a smaller margin that at $B = 0$) or the optical alignment thereby reducing the product $\eta_{\mathrm{optics}} \cdot \eta_{\mathrm{coupler}}$. We stress that this slight discrepancy between the predicted overall efficiency and the overall efficiency that matches best our experimental data has no impact on our readout fidelity analysis.

Supplementary Note 5.2. Calculation of the Readout Fidelity

The fidelity of the spin-readout is defined as

$$\mathcal{F}(t) = 1 - p_{\mathrm{bright}} \cdot e_{\mathrm{bright}}(t) - p_{\mathrm{dark}} \cdot e_{\mathrm{dark}}(t), \tag{2}$$

where in our experiments both p_{bright} and p_{dark} are approximately equal to 50%. The readout projects the spin into either the bright state or the dark state; the readout process records an outcome, either bright or dark. If the spin is projected into the bright (dark) state but readout as dark (bright) then the error is e_{bright} (e_{dark}). These error probabilities are time-dependent in that they depend on the duration of the readout pulse.

The errors in the readout have several origins. The bright spin state is correctly assigned if a photon is measured. Therefore, photon loss is an important source of readout error. The overall system efficiency η therefore contributes to e_{bright}. This source of error can be quantified by isolating this loss process in a simulation which takes the experimental

value of η but without back-action and without a spin-flip process. This results in $C(t)$, the cumulative distribution function for collecting a count as a function of time induced by a readout pulse starting at $t = 0$. In our experiments the error probability $e_{\rm bright} = 6.9\%$ at 3 ns, as shown in Fig. 2(d) in the main text. Another source of error for the bright state readout is a spin flip during the readout process: the QD can be projected into the bright state by the readout pulse but if it flips to the dark state before a photon is detected the spin is assigned incorrectly. These combined contributions to $e_{\rm bright}$ result in:

$$e_{\rm bright}(t) = 1 - C(t) + C(t) \cdot [1 - \exp(-t/\tau_{SF})] = 1 - C(t) \cdot \exp(-t/\tau_{SF}), \tag{3}$$

where τ_{SF} is the spin-flip time.

The dark state readout error also has two origins. First, the readout can project the spin into the dark state yet be recorded as the bright state should a laser photon leak into the collection channel and be detected. This error can be estimated and taken into account by measuring the count fraction $C_d(t)$ on turning off the QD, i.e. detuning the QD with respect to the readout laser (in practice via the gate voltage). Second, as for the bright state, a spin-flip can lead to an error: the readout can project the spin into the dark state yet be recorded as bright if a spin-flip from dark-to-bright state occurs followed by photon detection. The analysis of the second error is more complicated than that of the first. If the spin flips from the dark to the bright state, a photon can be emitted and counted. This takes place with the same time-dependence as $C(t)$, but shifted in time by the location in time of the spin-flip. This effect can be taken into account by a convolution of the shifted count fraction with the spin-flip probability. The combined readout error is therefore:

$$e_{dark}(t) = C_d(t) + \frac{1}{t} \int_0^t C(t-\tau) \cdot [1 - \exp(-t/\tau_{SF})] \cdot d\tau. \tag{4}$$

For a 3 ns readout pulse, the error probability $e_{\rm dark} = 2.6\%$. For short readout times, the error in reading out the state $e_{\rm bright}$ is high, as not enough time has elapsed to ensure that one of the QD photon is detected by the detector. For longer readout times, the probability of a spin-flip increases, and hence the probability of detecting a photon from the dark state via a spin flip to the bright state ($e_{\rm dark}$) increases. Hence, there is an optimal readout time for which the fidelity can be maximised. By plugging Eq. 3 and 4 into Eq. 2, we can calculate the fidelity of the readout as a function of the readout time; this is shown in Fig. 2(c) in the main text. We carry out this calculation on tuning the cavity to the higher-frequency trion and, separately, on tuning the cavity to the lower-frequency trion. The readout fidelity reaches 95.2% in 3 ns.

Supplementary Fig. 6 shows the dependence of the fidelity on the end-to-end efficiency (Supplementary Fig. 6 (a)), Purcell factor (Supplementary Fig. 6 (b)) and spin-flip time ((Supplementary Fig. 6 (c)). In Supplementary Fig. 6 when one of the parameters is varied, the others are set to match our present experimental conditions. However, for realistic improvements to all of these parameters simultaneously the readout can be significantly improved, as we now discuss.

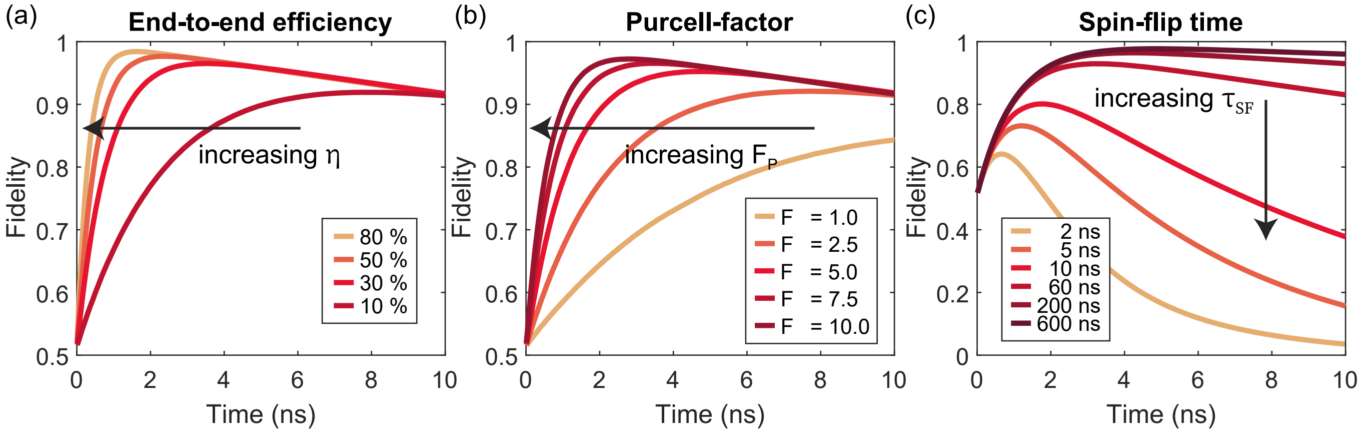

SUPPLEMENTARY FIG. 6: Simulation of the fidelity as a function of readout time for different **(a)** overall efficiencies, **(b)** Purcell factors and **(c)** spin-flip times. While one of the parameters is varied, the other two are set to the experimental conditions: $\eta = 25\%$, $F_P = 6.1$, and $\tau_{\rm SF} = 158$ ns. All simulations are performed at 6x the saturation power including 1.4% laser leakage at 3 ns readout duration.

Supplementary Note 5.3. Predictions for optimised system and Voigt geometry

Based on the success of the Monte-Carlo method in describing the experimental results, we can estimate the achievable fidelity for an optimised system as well as for single-shot readout in the Voigt geometry. We assume that the issue of imperfect laser suppression at the exact cavity resonance (Supplementary Note 4) can be overcome. We assume also that a QD can be selected with a larger optical dipole moment – we note that other QDs in the same sample show higher Purcell factors [10] than the QD used in these experiments – so that the Purcell factor can be increased from 6.1 to 12 without any modifications to the cavity. By reducing optical losses we estimate that $\eta_{\text{optics}} \cdot \eta_{\text{coupler}}$ can be increased from 55.2% to 90%. Finally, single-photon detectors with quantum efficiency $\eta_{\text{detector}} = 95\%$ (instead of 82%) are commercially available, and could also be used. These improvements would lead to $\eta = 76\%$ and would allow single-shot readout in less than 1 ns with a readout fidelity of 99.5%.

Although our readout speed is extremely fast, a key question is whether we can read the spin state fast enough to overcome the back-action in the Voigt geometry (in-plane magnetic field) as this is the configuration required for spin control. With $F_P = 12$, the branching ratio is 92.3%. In the optimised case ($\eta = 76\%$), we expect we can achieve single-shot readout with a fidelity of 89.9% below 1 ns, while for our present experimental conditions, single-shot readout should already be possible with a fidelity as high as 77.4% in 3 ns. These readout fidelities are extremely promising. Our approach can thus overcome a key outstanding challenge, namely combining spin control and spin readout in a single QD spin.

[1] Verberk, R., van Oijen, A. M. & Orrit, M. Simple model for the power-law blinking of single semiconductor nanocrystals. *Phys. Rev. B* **66**, 233202 (2002).

[2] Yilmaz, S. T., Fallahi, P. & Imamoglu, A. Quantum-dot-spin single-photon interface. *Phys. Rev. Lett.* **105**, 033601 (2010).

[3] Smith, J. M. *et al.* Voltage Control of the Spin Dynamics of an Exciton in a Semiconductor Quantum Dot. *Phys. Rev. Lett.* **94**, 197402 (2005).

[4] Dreiser, J. *et al.* Optical investigations of quantum dot spin dynamics as a function of external electric and magnetic fields. *Phys. Rev. B* **77**, 075317 (2008).

[5] Lu, C.-Y. *et al.* Direct measurement of spin dynamics in inas/gaas quantum dots using time-resolved resonance fluorescence. *Phys. Rev. B* **81**, 035332 (2010).

[6] Atatüre, M. *et al.* Quantum-Dot Spin-State Preparation with Near-Unity Fidelity. *Science* **312**, 551–553 (2006).

[7] Javadi, A. *et al.* Spin–photon interface and spin-controlled photon switching in a nanobeam waveguide. *Nat. Nanotechnol.* **13**, 398–403 (2018).

[8] Gaebel, T. *et al.* Stable single-photon source in the near infrared. *New J. Phys.* **6**, 98–98 (2004).

[9] Kuhlmann, A. V. *et al.* A dark-field microscope for background-free detection of resonance fluorescence from single semiconductor quantum dots operating in a set-and-forget mode. *Rev. Sci. Instrum.* **84**, 073905 (2013).

[10] Tomm, N. *et al.* A bright and fast source of coherent single photons. *Nat. Nanotechnol.* **16**, 399–403 (2021).
